# Supplementary material for: Analysis of PPARγ Signaling Activity in Psoriasis
Source: Int J Mol Sci. 2021 Aug 10;22(16):8603. doi: 10.3390/ijms22168603 (PMC8395241; doi:10.3390/ijms22168603)
Supplement: Supplementary file 1 [file ijms-22-08603-s001.zip › Supplemental materials_Analysis of PPARg signaling activity in psoriasis/Pathway models/Models images and html files/Model of PPARG related pathways in psoriasis (short version)/6436.html]

CXCL10


# Protein CXCL10

|  |  |
| --- | --- |
| URN | urn:agi-llid:3627 |
| Total Entities | 0 |
| Connectivity | 5018 |
| Name | CXCL10 |
| Description | chemokine (C-X-C motif) ligand 10 |
| Notes | This antimicrobial gene encodes a chemokine of the CXC subfamily and ligand for the receptor CXCR3. Binding of this protein to CXCR3 results in pleiotropic effects, including stimulation of monocytes, natural killer and T-cell migration, and modulation of adhesion molecule expression. [provided by RefSeq, Sep 2014] |
| Primary Cell Localization | Extracellular |
| Class | Ligand |
| ObjectType | Protein |

---

|  |  |
| --- | --- |
| Pathway | CXC Chemokine Receptor Signaling |
|  | Macrophage M1 Lineage |
|  | Nociception Expression Targets Signaling |
|  | CCR1 Expression Targets |
|  | Proteins Involved in Atherosclerosis |
|  | Chemokines Signaling in Atherosclerosis |
|  | Proteins Involved in Myocarditis |
|  | Proteins Involved in Psoriasis |
|  | Toll-like Receptors in beta-Cell |
|  | Proteins Involved in Diabetes Mellitus Type 1 |
|  | Proteins Involved in Diabetic Nephropathy |
|  | Proteins Involved in Hashimoto's Thyroiditis |
|  | Hashimoto's Thyroiditis |
|  | Proteins Involved in Graves Disease |
|  | Proteins Involved in Inflammatory Bowel Diseases |
|  | Leukocyte Migration toward the Endothelial Cell in Ulcerative Colitis |
|  | B-Cell Chronic Lymphocytic Leukemia |
|  | Mast-Cell Related Chemokines Signaling in Asthma |
|  | TLR2 Induced Synovial Fibroblast Activation in Rheumatoid Arthritis |
|  | Proteins Involved in Rheumatoid Arthritis |
|  | Proteins Involved in Systemic Scleroderma |
|  | Proteins Involved in Glioma |
|  | Proteins Involved in Neuroblastoma |
|  | Proteins Involved in Multiple Sclerosis |
|  | IL6/IL12 Signaling Activates Immune System in Multiple Sclerosis |
|  | Proteins Involved in Age-Related Macular Degeneration |
|  | Proteins with Altered Expression in Asthma |
|  | Proteins Involved in Chronic Obstructive Pulmonary Disease |
|  | Proteins Involved in Glomerulonephritis |
|  | CCL3 Expression Targets |
|  | CCL7 Expression Target |
|  | CCL2 Expression Targets |
|  | CSF2 -> NF-kB Expression Targets |
|  | CSF2 -> STAT Expression Targets |
|  | Elevated Receptors -> Expression Targets in Liver |
|  | EGFR -> Expression Targets in Skin |
|  | IFNB1/IFNR Expression Targets |
|  | IFNG/IFNR Expression Targets |
|  | IL13 Expression Targets |
|  | IL2 Expression Targets |
|  | Leptin -> STAT Expression Targets |
|  | Elevated Receptors -> Expression Targets in Lymph Node |
|  | GPCRs Family -> Expression Targets in Lymphoid System and Blood |
|  | Immunoglobulin Superfamily -> Expression Targets in Lymphoid System and Blood |
|  | Interleukin Receptors -> Expression Targets in Lymphoid System and Blood |
|  | CD40 -> Expression Targets in Thymus |
|  | CD40 -> Expression Targets in Lymph Node |
|  | Chemokine Receptor Family -> Expression Targets in Lymphoid System |
|  | AGT -> STAT Expression Targets |
|  | PRL/PRLR Expression Targets |
|  | PRL/GHR -> STAT Expression Targets |
|  | PRL/GHR -> NF/kB/ELK/SRF/MYC Expression Targets |
|  | Elevated Receptors -> Expression Targets in Respiratory System |
|  | TNF -> NF-kB Expression Targets |
|  | CD40LG -> STAT Expression Targets |
|  | CD40LG -> NF-kB/ELK/SRF -> CREB/NFATC Expression Targets |
|  | TNF -> STAT Expression Targets |
|  | TLR3 Expression Targets |
|  | TLR1 -> 2/6 Expression Targets |
|  | IL1B Expression Targets |
|  | TLR4 -> NF-kB/IRF Expression Targets |
|  | IL1A Expression Targets |
|  | Elevated Receptors -> Expression Targets in Kidney |
|  | VEGFA -> STAT Expression Targets |
|  | pathway 4 |
|  | pprarg neg, uknown targets, ps-positive |
|  | prarg neg,ukn expres targets, ps-positive |
|  | PPARG negative targets |
|  | pparg expr target\_regulators |
|  | Neighbors of disease exacerbation |
|  | Neighbors of tumor progression |
|  | Neighbors of cancer progression |
|  | Neighbors of inflammatory status |
|  | Neighbors of overall survival |
|  | Neighbors of disease severity |
|  | Neighbors of tumor size |
|  | Neighbors of mortality |
|  | Neighbors of survival rate |
|  | Neighbors of survival time |
|  | Neighbors of functional recovery |
|  | Neighbors of bacterial load |
|  | Neighbors of graft survival |
|  | Neighbors of therapeutic efficacy |
|  | Neighbors of lethality |
|  | Neighbors of tumor regression |
|  | Neighbors of recurrence-free survival |
|  | Neighbors of tumor response |
|  | Neighbors of macrophage count |
|  | Neighbors of long-term survival |
|  | Neighbors of treatment outcome |
|  | Neighbors of bone mass |
|  | Neighbors of body weight |
|  | Neighbors of microvessel density |
|  | Neighbors of tumor recurrence |
|  | Neighbors of neutrophil count |
|  | Neighbors of morbidity |
|  | Neighbors of fibrosis progression |
|  | Neighbors of exhaustion |
|  | Neighbors of adverse outcome |
|  | Neighbors of cancer-specific survival |
|  | Neighbors of apoptosis rate |
|  | Neighbors of shear stress |
|  | Neighbors of viral load |
|  | Neighbors of vascular leakage |
|  | Neighbors of blood glucose |
|  | Neighbors of ejection fraction |
|  | Neighbors of renal hypertrophy |
|  | Neighbors of T-cell count |
|  | Neighbors of glucose level |
|  | Neighbors of functional status |
|  | Neighbors of neurological outcome |
|  | Neighbors of monocyte count |
|  | Neighbors of disease exacerbation |
|  | Neighbors of tumor progression |
|  | Neighbors of cancer progression |
|  | Neighbors of inflammatory status |
|  | Neighbors of overall survival |
|  | Neighbors of disease severity |
|  | Neighbors of tumor size |
|  | Neighbors of mortality |
|  | Neighbors of survival rate |
|  | Neighbors of survival time |
|  | Neighbors of functional recovery |
|  | Neighbors of bacterial load |
|  | Neighbors of graft survival |
|  | Neighbors of therapeutic efficacy |
|  | Neighbors of lethality |
|  | Neighbors of tumor regression |
|  | Neighbors of recurrence-free survival |
|  | Neighbors of tumor response |
|  | Neighbors of macrophage count |
|  | Neighbors of long-term survival |
|  | Neighbors of treatment outcome |
|  | Neighbors of bone mass |
|  | Neighbors of body weight |
|  | Neighbors of microvessel density |
|  | Neighbors of tumor recurrence |
|  | Neighbors of neutrophil count |
|  | Neighbors of morbidity |
|  | Neighbors of fibrosis progression |
|  | Neighbors of exhaustion |
|  | Neighbors of adverse outcome |
|  | Neighbors of cancer-specific survival |
|  | Neighbors of apoptosis rate |
|  | Neighbors of shear stress |
|  | Neighbors of viral load |
|  | Neighbors of vascular leakage |
|  | Neighbors of blood glucose |
|  | Neighbors of ejection fraction |
|  | Neighbors of renal hypertrophy |
|  | Neighbors of T-cell count |
|  | Neighbors of glucose level |
|  | Neighbors of functional status |
|  | Neighbors of neurological outcome |
|  | Neighbors of monocyte count |
|  | Neighbors of cell development |
|  | Neighbors of immune response |
|  | Neighbors of cell population |
|  | Neighbors of T-cell development |
|  | Neighbors of inflammatory response |
|  | Neighbors of wound healing |
|  | Neighbors of angiogenesis |
|  | Neighbors of innate immune response |
|  | Neighbors of cell function |
|  | Neighbors of macrophage activation |
|  | Neighbors of cell formation |
|  | Neighbors of adaptive immune response |
|  | Neighbors of osteoclast development |
|  | Neighbors of epithelial to mesenchymal transition |
|  | Neighbors of immunity |
|  | Neighbors of cell proliferative response |
|  | Neighbors of chemotaxis |
|  | Neighbors of dendritic cell differentiation |
|  | Neighbors of cell survival |
|  | Neighbors of osteoclast differentiation |
|  | Neighbors of cell phenotype |
|  | Neighbors of T-cell proliferation |
|  | Neighbors of tissue remodeling |
|  | Neighbors of cell adhesion |
|  | Neighbors of T-cell activation |
|  | Neighbors of endothelial cell proliferation |
|  | Neighbors of fibrogenesis |
|  | Neighbors of tumor growth |
|  | Neighbors of dendritic cell development |
|  | Neighbors of tissue repair |
|  | Neighbors of cell count |
|  | Neighbors of cell infiltration |
|  | Neighbors of cell interaction |
|  | Neighbors of SMC proliferation |
|  | Neighbors of lumen formation |
|  | Neighbors of endothelial cell migration |
|  | Neighbors of lipid storage |
|  | Neighbors of cell damage |
|  | Neighbors of pregnancy |
|  | Neighbors of cell motility |
|  | Neighbors of bone resorption |
|  | Neighbors of monocyte migration |
|  | Neighbors of fibroblast proliferation |
|  | Neighbors of macrophage migration |
|  | Neighbors of microglial activation |
|  | Neighbors of neutrophil recruitment |
|  | Neighbors of regeneration |
|  | Neighbors of cell migration |
|  | Neighbors of cancer growth |
|  | Neighbors of stem cell proliferation |
|  | Neighbors of smooth muscle cell migration |
|  | Neighbors of hepatic regeneration |
|  | Neighbors of transendothelial migration |
|  | Neighbors of phagocytosis |
|  | Neighbors of immune system activation |
|  | Neighbors of leukocyte migration |
|  | Neighbors of leukocyte recruitment |
|  | Neighbors of osteoclast formation |
|  | Neighbors of monocyte recruitment |
|  | Neighbors of cell invasion |
|  | Neighbors of T-cell function |
|  | Neighbors of immune system function |
|  | Neighbors of transcription activation |
|  | Neighbors of T-cell response |
|  | Neighbors of tumor immunity |
|  | Neighbors of hepatocyte proliferation |
|  | Neighbors of cell transdifferentiation |
|  | Neighbors of neuroprotection |
|  | Neighbors of stem cell differentiation |
|  | Neighbors of endothelial cell function |
|  | Neighbors of neuronal death |
|  | Neighbors of cell growth |
|  | Neighbors of ROS generation |
|  | Neighbors of epithelial cell proliferation |
|  | Neighbors of first trimester pregnancy |
|  | Neighbors of cancer cell growth |
|  | Neighbors of sensitization |
|  | Neighbors of hemato-encephalic barrier |
|  | Neighbors of macrophage function |
|  | Neighbors of cellular immune response |
|  | Neighbors of colony formation |
|  | Neighbors of macrophage differentiation |
|  | Neighbors of cell differentiation |
|  | Neighbors of stem cell migration |
|  | Neighbors of life span |
|  | Neighbors of psoriasis |
|  | Neighbors of inflammatory disease |
|  | Neighbors of rheumatoid arthritis |
|  | Neighbors of atherosclerosis |
|  | Neighbors of fibrosis |
|  | Neighbors of colitis |
|  | Neighbors of arthritis |
|  | Neighbors of inflammatory bowel disease |
|  | Neighbors of injury |
|  | Neighbors of atherogenesis |
|  | Neighbors of vascular remodeling |
|  | Neighbors of metastasis |
|  | Neighbors of carcinogenesis |
|  | Neighbors of multiple sclerosis |
|  | Neighbors of chronic inflammation |
|  | Neighbors of inflammation |
|  | Neighbors of tumor microenvironment |
|  | Neighbors of pathological angiogenesis |
|  | Neighbors of obesity |
|  | Neighbors of vascular disease |
|  | Neighbors of diabetes mellitus |
|  | Neighbors of asthma |
|  | Neighbors of pneumonia |
|  | Neighbors of hyperplasia |
|  | Neighbors of osteoarthritis |
|  | Neighbors of liver fibrosis |
|  | Neighbors of chronic obstructive pulmonary disease |
|  | Neighbors of myocardial infarction |
|  | Neighbors of leukocyte infiltration |
|  | Neighbors of reperfusion injury |
|  | Neighbors of airway inflammation |
|  | Neighbors of hepatitis |
|  | Neighbors of insulin resistance |
|  | Neighbors of infection |
|  | Neighbors of vascular injury |
|  | Neighbors of renal injury |
|  | Neighbors of macrophage infiltration |
|  | Neighbors of liver injury |
|  | Neighbors of neuroinflammation |
|  | Neighbors of sepsis |
|  | Neighbors of death |
|  | Neighbors of type 1 diabetes |
|  | Neighbors of encephalomyelitis |
|  | Neighbors of autoimmune disease |
|  | Neighbors of dermatitis |
|  | Neighbors of neutrophil infiltration |
|  | Neighbors of diabetic nephropathy |
|  | Neighbors of lung injury |
|  | Neighbors of immunopathology |
|  | Neighbors of systemic sclerosis |
|  | Neighbors of hepatocellular carcinoma |
|  | Neighbors of autoimmunity |
|  | Neighbors of acute lung injury |
|  | Neighbors of gastric cancer |
|  | Neighbors of atherosclerotic plaque |
|  | Neighbors of experimental autoimmune encephalomyelitis |
|  | Neighbors of colorectal cancer |
|  | Neighbors of cardiac remodeling |
|  | Neighbors of breast cancer |
|  | Neighbors of ischemia |
|  | Neighbors of systemic lupus erythematosus |
|  | Neighbors of melanoma |
|  | Neighbors of collagen-induced arthritis |
|  | Neighbors of cartilage degeneration |
|  | Neighbors of interstitial fibrosis |
|  | Neighbors of kidney disease |
|  | Neighbors of heart failure |
|  | Neighbors of lung disease |
|  | Neighbors of pulmonary fibrosis |
|  | Neighbors of cardiovascular disease |
|  | Neighbors of stroke |
|  | Neighbors of cancer |
|  | Neighbors of cerebral ischemia |
|  | Neighbors of type 2 diabetes |
|  | Neighbors of neoplasm |
|  | Neighbors of vasculitis |
|  | Neighbors of respiratory hypersensitivity |
|  | Neighbors of myocardial fibrosis |
|  | Neighbors of synovitis |
|  | Neighbors of rupture |
|  | Neighbors of endothelial cell dysfunction |
|  | Neighbors of liver disease |
|  | Neighbors of osteolysis |
|  | Neighbors of ovarian cancer |
|  | Neighbors of hypertension |
|  | Neighbors of intimal hyperplasia |
|  | Neighbors of allograft rejection |
|  | Neighbors of proteinuria |
|  | Neighbors of epithelial cell |
|  | Neighbors of keratinocyte |
|  | Neighbors of vascular smooth muscle cell |
|  | Neighbors of umbilical vein endothelial cell |
|  | Neighbors of monocyte |
|  | Neighbors of endometrial stromal cell |
|  | Neighbors of alveolar macrophage |
|  | Neighbors of synoviocyte |
|  | Neighbors of macrophage |
|  | Neighbors of adipocyte |
|  | Neighbors of activated monocyte |
|  | Neighbors of fibroblast |
|  | Neighbors of airway epithelial cell |
|  | Neighbors of PBMC |
|  | Neighbors of stromal cell |
|  | Neighbors of airway smooth muscle cell |
|  | Neighbors of osteoblast |
|  | Neighbors of pneumocyte |
|  | Neighbors of endometrium cell |
|  | Neighbors of microglia |
|  | Neighbors of trophoblast |
|  | Neighbors of intestine epithelium cell |
|  | Neighbors of mononuclear cell |
|  | Neighbors of hepatic stellate cell |
|  | Neighbors of hepatocyte |
|  | Neighbors of decidual cell |
|  | Neighbors of kidney mesangial cell |
|  | Neighbors of endothelial cell |
|  | Neighbors of dendritic cell |
|  | Neighbors of Kuppfer cell |
|  | Neighbors of proadipocyte |
|  | Neighbors of kidney tubule cell |
|  | Neighbors of inflammatory cell |
|  | Neighbors of bone marrow macrophage |
|  | Neighbors of peritoneal macrophage |
|  | Neighbors of osteoclast |
|  | Neighbors of neutrophil |
|  | Neighbors of smooth muscle myocyte |
|  | Neighbors of astrocyte |
|  | Neighbors of bone marrow derived dendritic cell |
|  | Neighbors of lung fibroblast |
|  | Neighbors of activated T-cell |
|  | Neighbors of capillary endothelial cell |
|  | Neighbors of immunocompetent cell |
|  | Neighbors of granulocyte |
|  | Neighbors of cardiac myocyte |
|  | Neighbors of cancer-associated fibroblast |
|  | Neighbors of myeloid cell |
|  | Neighbors of mesenchymal stroma cell |
|  | Neighbors of corneal epithelial cell |
|  | Neighbors of stem cell |
|  | Neighbors of tumor infiltrating macrophage |
|  | Neighbors of sinusoidal cell |
|  | Neighbors of B-cell |
|  | Neighbors of leukocyte |
|  | Neighbors of insulin-secreting cell |
|  | Neighbors of T-cell |
|  | Neighbors of M1 macrophage |
|  | Neighbors of myofibroblast |
|  | Neighbors of pancreatic stellate cell |
|  | Neighbors of mast cell |
|  | Neighbors of bone marrow stromal cell |
|  | Neighbors of spleen cell |
|  | Neighbors of brain capillary endothelial cell |
|  | Neighbors of neuroglia |
|  | Neighbors of thyroid epithelial cell |
|  | Neighbors of hematopoietic cell |
|  | Neighbors of adipose macrophage |
|  | Neighbors of podocyte |
|  | Neighbors of conventional dendritic cell |
|  | Neighbors of pericyte |
|  | Neighbors of antigen-presenting cell |
|  | Neighbors of epidermis cell |
|  | Neighbors of bone marrow derived mast cell |
|  | Neighbors of kidney cell |
|  | Neighbors of syncytiotrophoblast |
|  | Neighbors of lymphocyte |
|  | Neighbors of Langerhans cell |
|  | Neighbors of decidual natural killer cell |
|  | Neighbors of thymic epithelial cell |
|  | Neighbors of brain cell |
|  | Neighbors of M2 macrophage |
|  | Neighbors of lymphoid cell |
|  | Neighbors of retina cell |
|  | Neighbors of blood cell |
|  | Neighbors of peripheral lymphocyte |
|  | Neighbors of activated B-cell |
|  | Neighbors of lung |
|  | Neighbors of liver |
|  | Neighbors of kidney |
|  | Neighbors of skin |
|  | Neighbors of intestine |
|  | Neighbors of lymph node |
|  | Neighbors of colon |
|  | Neighbors of airway |
|  | Neighbors of blood vessel |
|  | Neighbors of heart |
|  | Neighbors of gastrointestinal tract |
|  | Neighbors of microvessel |
|  | Neighbors of bone |
|  | Neighbors of placenta |
|  | Neighbors of synovial membrane |
|  | Neighbors of retina |
|  | Neighbors of brain |
|  | Neighbors of islets of Langerhans |
|  | Neighbors of joint |
|  | Neighbors of pancreas |
|  | Neighbors of central nervous system |
|  | Neighbors of kidney glomerulus |
|  | Neighbors of immune system |
|  | Neighbors of spleen |
|  | Neighbors of vein |
|  | Neighbors of glands |
|  | Neighbors of eye |
|  | Neighbors of myelin sheath |
|  | Neighbors of bladder |
|  | Neighbors of trachea |
|  | Neighbors of mediastinal lymph node group |
|  | Neighbors of hair follicle |
|  | Neighbors of NF-kB family |
|  | Neighbors of oxidized LDL |
|  | Neighbors of JNK |
|  | Neighbors of IL1 family |
|  | Neighbors of PPAR |
|  | Neighbors of Jun/Fos |
|  | Neighbors of NFKBI |
|  | Neighbors of PKC |
|  | Neighbors of PI3K |
|  | Neighbors of STAT family |
|  | Neighbors of mitogen-activated protein kinase |
|  | Neighbors of IFNAR ligand |
|  | Neighbors of TLR |
|  | Neighbors of cytokine |
|  | Neighbors of interferon |
|  | Neighbors of interleukin |
|  | Neighbors of JAK |
|  | Neighbors of MEK1/2 |
|  | Neighbors of PDGF |
|  | Neighbors of Ras GTPase |
|  | Neighbors of ERK1/2 |
|  | Neighbors of growth factor |
|  | Neighbors of PKA |
|  | Neighbors of chorionic gonadotropin |
|  | Neighbors of IKBK |
|  | Neighbors of adipokine |
|  | Neighbors of protein tyrosine kinase |
|  | Neighbors of IgG |
|  | Neighbors of AMPK |
|  | Neighbors of LXR |
|  | Neighbors of p38 |
|  | Neighbors of inflammatory cytokine |
|  | Neighbors of IL23 |
|  | Neighbors of T-cell receptor |
|  | Neighbors of histone deacetylase |
|  | Neighbors of IL1R |
|  | Neighbors of IL12 |
|  | Neighbors of prostaglandin-endoperoxide synthase |
|  | Neighbors of fibrinogen |
|  | Neighbors of aldehyde reductase |
|  | Neighbors of mitogen-activated protein kinase kinase |
|  | Neighbors of non-coding RNA |
|  | Neighbors of histone H3 |
|  | Neighbors of SAPK |
|  | Neighbors of AHR subfamily |
|  | Neighbors of CD3 complex |
|  | Neighbors of TLR1/2 heterodimer |
|  | Neighbors of histone |
|  | comon ps\_pos, pprarg\_neg targets |
|  | Model of PPARG signaling in psoriasis |
|  | PPARG negative regulators and targets |
|  | Model of PPARG related pathways in psoriasis (short version) |
|  | New Pathway (4) |
|  | IFN signaling in alveolar cells |
|  | Overview. Infectious and parasitic diseases (chapter 2) |
|  | 3\_Leukocyte migration toward the endothelial cells in the intestine microvasculature |
|  | drug-target moltransport |

---

|  |  |
| --- | --- |
| Group | Proteins Involved in Graves Ophthalmopathy |

---

|  |  |
| --- | --- |
| MedScan ID | 3627 |

---

|  |  |
| --- | --- |
| LocusLink ID | 3627 |
|  | 15945 |
|  | 245920 |

---

|  |  |
| --- | --- |
| Alias | Interferon-gamma induced protein 10 |
|  | IFI10 |
|  | SZ10\_HUMAN |
|  | IP10 |
|  | interferon-inducible protein-10 |
|  | C-X-C motif chemokine ligand 10 |
|  | crg-2 |
|  | CXCL10 |
|  | interferon-inducible cytokine IP-10 |
|  | CXCL10 chemokine |
|  | protein 10 from interferon (gamma)-induced cell line |
|  | small inducible cytokine B subfamily (Cys-X-Cys) member 10 |
|  | SCYB10 |
|  | interferon activated gene 10 |
|  | gIP-10 |
|  | chemokine CXCL10 |
|  | IFN-inducible protein 10 |
|  | SZ10 |
|  | Interferon-gamma-induced protein-10 |
|  | OTTMUSP00000036425 |
|  | gIP X |
|  | IFN-gamma-inducible protein 10 |
|  | interferon-inducible cytokine IP X |
|  | interferon-gamma induced protein CRG-2 |
|  | C-X-C motif chemokine 10 |
|  | CXC ligand 10 |
|  | chemokine (C-X-C motif) ligand 10 |
|  | gamma IP10 |
|  | Interferon-gamma-induced protein 10 |
|  | 10 kDa interferon gamma-induced protein |
|  | gamma interferon inducible protein 10 |
|  | Small inducible cytokine B10 |
|  | chemokine CXC ligand 10 |
|  | IFNG-inducible protein 10 |
|  | interferon-gamma inducible protein-10 |
|  | CXC chemokine ligand 10 |
|  | INP10 |
|  | C7 |
|  | IP-10 |
|  | mob-1 |
|  | small inducible cytokine subfamily B (Cys-X-Cys), member 10 |
|  | small-inducible cytokine B10 |
|  | gamma-IP10 |
|  | small inducible cytokine B subfamily (Cys-X-Cys), member 10 |
|  | interferon-inducible protein 10 |
|  | protein Mob-1 |
|  | crg II |
|  | interferon-gamma-induced protein CRG-2 |

---

|  |  |
| --- | --- |
| GO ID | 0048248 |
|  | 0008603 |
|  | 0042056 |
|  | 0008009 |
|  | 0008201 |
|  | 0005102 |
|  | 0007186 |
|  | 0010818 |
|  | 0007189 |
|  | 0061844 |
|  | 0008015 |
|  | 0007166 |
|  | 0007267 |
|  | 0034605 |
|  | 0071222 |
|  | 0070098 |
|  | 0006935 |
|  | 0019221 |
|  | 0051607 |
|  | 0042118 |
|  | 0006955 |
|  | 0006954 |
|  | 0030595 |
|  | 0007517 |
|  | 0016525 |
|  | 0045662 |
|  | 1901740 |
|  | 0030593 |
|  | 0050918 |
|  | 2000406 |
|  | 0008284 |
|  | 0090026 |
|  | 0051281 |
|  | 0045944 |
|  | 0010819 |
|  | 0042981 |
|  | 0042127 |
|  | 1901509 |
|  | 0045859 |
|  | 0010996 |
|  | 0009409 |
|  | 0010332 |
|  | 0033280 |
|  | 0007165 |
|  | 0009897 |
|  | 0005576 |
|  | 0005615 |
|  | 0005125 |
|  | 0006952 |
|  | 0030335 |
|  | 0002690 |
|  | 0009617 |
|  | 0010469 |
|  | 0032496 |
|  | 0009615 |
|  | 0005623 |
|  | 0009306 |
|  | 0030816 |
|  | 0006928 |
|  | 0043950 |
|  | 0045236 |

---

|  |  |
| --- | --- |
| KEGG ID | hsa:3627 |
|  | mmu:15945 |
|  | rno:245920 |

---

|  |  |
| --- | --- |
| Organism | Homo sapiens {Organism urn:agi-taxid:9606} |
|  | Mus musculus {Organism urn:agi-taxid:10090} |
|  | Rattus norvegicus {Organism urn:agi-taxid:10116} |
|  | Mus musculus |
|  | Homo sapiens |
|  | Rattus norvegicus |

---

|  |  |
| --- | --- |
| Mouse chromosome position | 5 46.57 cM |
|  | 5 53.0 cM |

---

|  |  |
| --- | --- |
| OMIM ID | 147310 |

---

|  |  |
| --- | --- |
| Rat chromosome position | 14p22 |

---

|  |  |
| --- | --- |
| Hugo ID | 10637 |
|  | HGNC:10637 |

---

|  |  |
| --- | --- |
| Human chromosome position | 4q21.1 |
|  | 4q21 |

---

|  |  |
| --- | --- |
| Swiss-Prot Accession | A0A024RDA4 |
|  | P02778 |
|  | P02778.2 |
|  | P17515 |
|  | Q548V9 |
|  | P17515.1 |
|  | P48973 |
|  | P48973.2 |
|  | Q96QJ5 |
|  | P70538 |
|  | Q6GTC7 |
|  | Q3U6X5 |
|  | Q3UAS6 |
|  | Q3UCV1 |
|  | Q3UK71 |

---

|  |  |
| --- | --- |
| PIR ID | A03243 |
|  | A45492 |

---

|  |  |
| --- | --- |
| GenBank ID | NC\_000004 |
|  | NM\_001565 |
|  | NP\_001556 |
|  | AC112719 |
|  | CH471057 |
|  | EAX05772 |
|  | EAX05773 |
|  | AA321649 |
|  | AA505379 |
|  | BC010954 |
|  | AAH10954 |
|  | BF056337 |
|  | X02530 |
|  | CAA26370 |
|  | P02778 |
|  | NC\_000071 |
|  | NM\_021274 |
|  | NP\_067249 |
|  | AC109603 |
|  | AC122365 |
|  | CH466617 |
|  | EDL05256 |
|  | L07417 |
|  | AAA75249 |
|  | M86830 |
|  | AF227743 |
|  | AAG10400 |
|  | AJ243095 |
|  | AK144279 |
|  | BAE25810 |
|  | AK146144 |
|  | BAE26931 |
|  | AK150380 |
|  | BAE29511 |
|  | AK150765 |
|  | BAE29832 |
|  | AK150987 |
|  | AK151210 |
|  | BAE30205 |
|  | AK151248 |
|  | BAE30238 |
|  | AK151415 |
|  | BAE30380 |
|  | AK151534 |
|  | BAE30481 |
|  | AK152568 |
|  | BAE31320 |
|  | AK152814 |
|  | BAE31517 |
|  | AK152838 |
|  | AK152924 |
|  | BAE31599 |
|  | AK153181 |
|  | BAE31783 |
|  | AK156907 |
|  | AK157130 |
|  | BAE33973 |
|  | AK157139 |
|  | BAE33975 |
|  | AK157589 |
|  | BAE34130 |
|  | AK157678 |
|  | BAE34149 |
|  | AK172540 |
|  | BAE43058 |
|  | BC030067 |
|  | AAH30067 |
|  | BC057150 |
|  | BY165450 |
|  | M33266 |
|  | AAA02968 |
|  | M86829 |
|  | AAA21489 |
|  | P17515 |
|  | NC\_005113 |
|  | NM\_139089 |
|  | NP\_620789 |
|  | AC\_000082 |
|  | AAHX01078298 |
|  | AC113621 |
|  | CH474060 |
|  | EDL88637 |
|  | BC058444 |
|  | AAH58444 |
|  | FQ225009 |
|  | FQ225217 |
|  | FQ225221 |
|  | FQ225582 |
|  | FQ225670 |
|  | FQ226479 |
|  | FQ231406 |
|  | FQ232883 |
|  | FQ233441 |
|  | FQ233623 |
|  | FQ233884 |
|  | FQ234688 |
|  | U17035 |
|  | AAB60485 |
|  | U22520 |
|  | AAC52811 |
|  | P48973 |
|  | DQ892786 |
|  | AMYH02009504 |
|  | AC\_000027 |
|  | ABM87032 |
|  | AC\_000136 |
|  | AABR06077780 |
|  | DQ896033 |
|  | AAHY01046867 |
|  | ABBA01063355 |
|  | NC\_018915 |
|  | ABM83712 |
|  | NT\_016354 |
|  | NW\_922162 |
|  | NW\_001838915 |
|  | AC\_000047 |
|  | NT\_109320 |
|  | NW\_001030791 |
|  | AK152234 |
|  | Q3U6X5 |
|  | Q3UAS6 |
|  | Q3UCV1 |
|  | Q3UK71 |
|  | Q548V9 |
|  | NW\_001084690 |
|  | NW\_047423 |
|  | NT\_039339 |

---

|  |  |
| --- | --- |
| Swiss-Prot ID | CXL10\_HUMAN |
|  | CXL10\_MOUSE |
|  | CXL10\_RAT |
|  | Q548V9\_MOUSE |

---

|  |  |
| --- | --- |
| Cell Localization | Secreted |
|  | Extracellular |

---

|  |  |
| --- | --- |
| Ensembl ID | ENSG00000169245 |
|  | ENSP00000305651.1 |
|  | ENST00000306602.3 |
|  | ENSMUSG00000034855 |
|  | ENSMUSP00000047646.6 |
|  | ENSMUST00000038816.12 |
|  | ENSRNOG00000022256 |
|  | ENSRNOP00000003649.4 |
|  | ENSRNOT00000003075.7 |
|  | ENSRNOP00000003649 |
|  | ENST00000306602 |
|  | ENSMUST00000038816 |
|  | ENST00000306602.2 |
|  | ENSRNOT00000003075 |
|  | ENSMUSP00000047646 |
|  | ENSP00000305651 |

---

|  |  |
| --- | --- |
| MGI ID | MGI:1352450 |
|  | 1352450 |

---

|  |  |
| --- | --- |
| RGD ID | 620209 |

---

|  |  |
| --- | --- |
| Unigene ID | Mm.877 |
|  | Hs.632586 |
|  | Rn.10584 |

---

|  |  |
| --- | --- |
| Homologene ID | 1203 |

---

|  |  |
| --- | --- |
| Shape | Rhomb |

---

|  |  |
| --- | --- |
| IPI ID | IPI00022448 |
|  | IPI00109915 |
|  | IPI00231025 |
|  | IPI00872038 |
|  | IPI00880488 |

---
